# *‘Jeotgalicoccus saudimassiliensis’* sp. nov., a new bacterial species isolated from air samples in the urban environment of Makkah, Saudi Arabia

**DOI:** 10.1016/j.nmni.2016.12.002

**Published:** 2016-12-09

**Authors:** A. Papadioti, E.I. Azhar, F. Bibi, A. Jiman-Fatani, S.M. Aboushoushah, M. Yasir, D. Raoult, E. Angelakis

**Affiliations:** 1) Unité de Recherche sur les Maladies Infectieuses et Tropicales Emergentes: URMITE CNRS-IRD 198 UMR 6236, Aix Marseille Université, Faculté de Médecine, Marseille, France; 2) Special Infectious Agents Unit, King Fahd Medical Research Center, Jeddah, Saudi Arabia; 3) Department of Medical Laboratory Technology, Faculty of Applied Medical Sciences, Jeddah, Saudi Arabia; 4) Department of Medical Microbiology and Parasitology, Faculty of Medicine, King Abdulaziz University, Jeddah, Saudi Arabia

**Keywords:** Air isolates, culturomics, *‘Jeotgalicoccus saudimassiliensis’*, Saudi Arabia

## Abstract

We report here the main characteristics of *‘Jeotgalicoccus saudimassiliensis’* strain 13MG44_air^T^ (CSUR P1221), a new species of the *Jeotgalicoccus* genus that was isolated from air samples in the city environment of Makkah, Saudi Arabia, during the pilgrim period of Hajj 2012.

As a part of a wider culturomics [1] and metagenomics study [2] in Saudi Arabia, we isolated a new bacterium, strain 13MG44_air^T^, from two air samples in the urban environment of Makkah, Saudi Arabia, during the pilgrim period of Hajj 2012. For each air sample, a volume of 1 m^3^ was collected with a FCC-IV biological air sampler (AES Laboratories, Combourg, France) mounted with a nutrient agar plate containing the antifungal agent amphotericin (Majed Al-Buqami Co. BMC, Riyadh, Saudi Arabia) according to the manufacturer’s instructions. The strain 13MG44_air^T^ was cultured in 5% sheep’s blood–enriched Columbia agar (bioMérieux, Marcy l’Etoile, France) for 2 days in an aerobic atmosphere at 37°C. Growth was observed in the range 0 to 15% NaCl with an optimum at 5% NaCl in aerobic conditions, and no growth occurred in anaerobic conditions. Strain 13MG44_air^T^ colonies on Columbia agar were opaque, round and white-grey in color, and they varied between 1.2 to 2.7 mm in diameter. The strain 13MG44_air^T^ is a Gram-positive, aerobic, nonmotile, catalase- and oxidase-positive, coccus-shaped organism. No identification was obtained for the strain 13MG44_air^T^ using our systematic matrix-assisted laser desorption/ionization time-of-flight mass spectrometry (MALDI-TOF MS) screening on a MicroFlex spectrometer (Bruker Daltonics, Bremen, Germany).

The complete 16S rRNA gene was sequenced using fD1-rP2 primers as previously described and a 3130-XL sequencer (Applied Biosciences, Saint Aubin, France) [3]. The strain 13MG44_air^T^ exhibited a 98.5% sequence similarity with *Jeotgalicoccus psychrophilus* (JQ266291) which was the phylogenetically closest species with standing in nomenclature (Fig. 1). Consequently it putatively classifies the strain 13MG44_air^T^ as a new member of the genus *Jeotgalicoccus* within the family *Staphylococcaceae* in the phylum *Firmicutes.* The genus *Jeotgalicoccus* was first described by Yoon *et al.* in 2003 by the isolation of *Jeotgalicoccus halotolerans* and *Jeotgalicoccus psychrophilus* in a traditional Korean fermented seafood [4]. The genus *Jeotgalicoccus* was later emended by Liu *et al.* by the isolation of *Jeotgalicoccus nanhaiensis* from intertidal sediment [5]. *Jeotgalicoccus* species were detected as inhabitants of a bovine teat canal [6], and recently *Jeotgalicoccus aerolatus* was isolated from bioaerosol samples from a poultry-fattening industry and *Jeotgalicoccus coquinae* was isolated from coquina, a food supplement for female ducks used in a duck-fattening farm [7].

Strain 13MG44_air^T^ exhibited a 16S rRNA gene sequence divergence of >1.3% with *J. psychrophilus,* the closest related species with standing in nomenclature, which classifies it as a new representative of the *Jeotgalicoccus* genus isolated from air samples in the urban environment of Makkah. As a result, we propose the creation of *‘Jeotgalicoccus saudimassiliensis’* sp. nov., and the strain 13MG44_air as the type strain.

## MALDI-TOF MS spectrum

The MALDI-TOF MS spectrum of strain 13MG44_air^T^ is available online (http://www.mediterranee-infection.com/article.php?laref=256&titre=urms-database).

## Nucleotide sequence accession number

The 16S rRNA gene sequence of strain 13MG44_air^T^ was deposited in GenBank under accession number HG931342.1.

## Deposit in a culture collection

Strain 13MG44_air^T^ was deposited in the Collection de Souches de l’Unité des Rickettsies (CSUR, WDCM 875) under number P1221.

## Figures and Tables

**FIG. 1:**
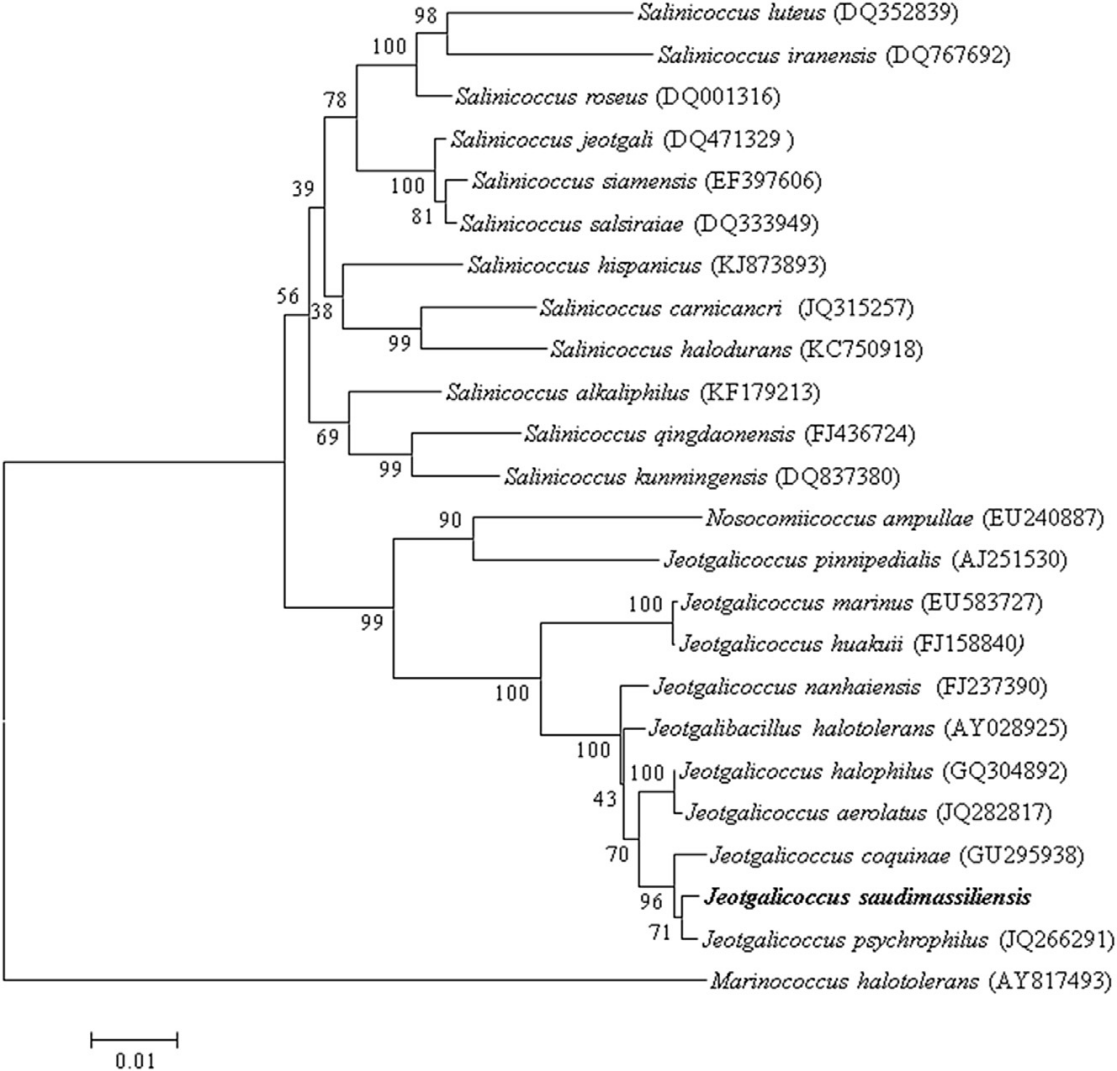
Phylogenetic tree highlighting position of *‘Jeotgalicoccus saudimassiliensis’* relative to other phylogenetically close members of *Jeotgalicoccus* genus. Numbers at nodes are percentages of bootstrap values obtained by repeating analysis 500 times to generate majority consensus tree. Scale bar represents 1% nucleotide sequence divergence.
